# Connective Tissue Growth Factor: A Key Factor Among Mediators of Tissue Fibrosis

**DOI:** 10.18502/jovr.v17i4.12294

**Published:** 2022-11-29

**Authors:** Nader Sheibani

**Affiliations:** ^1^Department of Ophthalmology and Visual Sciences, University of Wisconsin School of Medicine and Public Health, Madison, WI, USA; ^2^Department of Cell and Regenerative Biology, University of Wisconsin School of Medicine and Public Health, Madison, WI, USA; ^3^McPherson Eye Research Institute, University of Wisconsin School of Medicine and Public Health, Madison, WI, USA; ^4^Department of Biomedical Engineering, University of Wisconsin, Madison, WI, USA; ^6^https://orcid.org/0000-0003-2723-9217

Fibrosis is the increased accumulation of fibrous connective tissue in and around tissues with inflammation or damage, which initiates irreversible scar formation.^[1]^ It is a main reason for the development of adverse events in many chronic inflammatory diseases. Despite many efforts in the development of therapeutics for fibrosis, success has been very limited. However, recent advancements in single cell omics' tools are helping to delineate the molecular and cellular mechanisms that contribute to fibrosis and provide the opportunity for more targeted new approaches for treatment of fibrosis.^[2]^ Fibrosis contributes to disorders in many tissues and organs including liver and heart dysfunction, rheumatoid arthritis, diabetic nephropathy and retinopathy, age-related macular degeneration, corneal wound healing, and glaucoma.^[1,3]^ However, the underlying cellular and molecular mechanisms may vary in different organs, and the commonality among these diverse fibrotic diseases is not fully delineated. Connective tissue growth factor (CTGF) has been recognized as an important mediator of fibrosis and scar formation.^[4,5,6,7,8,9,10,11]^ A recent study utilizing computational strategies identified CTGF as the common key molecule regulating fibrogenesis among eight different fibrotic diseases including ocular fibrosis.^[12]^ Thus, many efforts have focused on interfering with the expression and activity of CTGF, such as function blocking antibodies,^[13,14,15,16]^ siRNA,^[17,18]^ shRNA,^[19]^ microRNA,^[20,21]^ Yap/Taz inhibitor,^[22,23,24]^ and most recently CRISPER-Cas9 system^[25]^ to mitigate fibrosis in different cells, tissues, and organs including the eye. Fibrosis is a complex and active process, which is impacted by various cell types, differentiation and signaling pathways, genes, and crosstalk.^[1,26]^

The deposition of excessive extracellular matrix proteins by myofibroblasts is common to many fibrotic conditions.^[27,28,29]^ Perivascular supporting cells, which are also tissue resident mesenchymal stem cells, are recently recognized as being the cells primarily involved in fibrotic responses in many organs and tissues.^[30]^ We have noted that retinal and choroidal perivascular supporting cells express higher levels of CTGF compared to retinal or choroidal endothelial cells but lower than microglial and retinal pigment epithelial cells (our unpublished observations). The high levels of CTGF in microglial cells decreases while that of pericytes increases in response to high glucose conditions,^[31]^ and may drive migration and/or loss of perivascular cells from retinal blood vessels during diabetes.^[32]^ Immune cells, such as macrophages, mediate inflammatory responses that are integrated with other pro-fibrotic processes.^[29,31,33,34,35]^ However, the details of these interactions remain poorly understood, but single-cell genomic, proteomic, and transcriptomic techniques are further unraveling the specific cell subtypes and their interactions in pathophysiology of fibrosis in various tissues.

In terms of signaling pathways, TGF-β has been extensively recognized as key mediator of pro-fibrotic activity.^[3]^ Since the protein is made as an inactive precursor and deposited in the extracellular matrix (ECM), its mechanisms of activation by αvβ6 integrin, autotaxin, and galectin-3 have been targeted for potential therapeutics.^[1,3]^ Unfortunately, targeting these mechanisms of TGF-β activation have been ineffective in treating and/or preventing fibrosis, especially in idiopathic pulmonary fibrosis.^[26]^ These failures have been attributed, at least in part, to normal physiological functions of TGF-β, and its systemic inactivation could have many adverse effects. Thus, not only the identification of pathways involved are important but determining their hierarchy is essential to their successful targeting for therapeutic intentions. CTGF is a downstream target of TGF-β in tissue remodeling and ECM synthesis. It is also proinflammatory and is recognized as a common key driver of fibrosis in various fibrotic disorders.^[12]^ Thus, focusing on the role of inflammatory cells and their mediators in modulation of innate immune cell responses in driving fibrosis could be beneficial.^[26]^ Revealing the identity, organization, and mechanical properties of the ECM environment in fibrosis could also provide additional unique clues to the mechanisms that drives inappropriate activation of target cells.

Trabeculectomy is a surgical treatment to lower intraocular pressure in glaucoma patients. However, excess scaring leads to failure of the filtering bleb and adversely affect the treatment outcome.^[36,37]^ It is well-established that CTGF expression is significantly upregulated during this fibrotic response and targeting CTGF using various modalities, as stated above, are shown to prevent fibrotic signaling including proliferation, ECM deposition, and proinflammatory activity in fibroblasts and retinal pigment epithelial cells, and in *in vivo* preclinical models. In a manuscript in this issue of *JOVR*, Hassanpour and colleagues^[38]^ demonstrate subconjunctival delivery of anti-CTGF is efficacious in prolonging the bleb life in a rabbit model of trabeculectomy. They did a careful analysis of the microstructural features of the bleb areas and provide histological proof on the inhibition or reduction of fibrosis in anti-CTGF treated trabeculectomy blebs. The changes in response to anti-CTGF were comparable with those noted in mitomycin C (MMC) treatment. They also showed MMC treatment was concomitant with decreased production of CTGF, likely as a result of the inhibition of cell proliferation by MMC. Growth arrested cells, a limitation with use of MMC, are however metabolically active and could release other factors, which could exacerbate fibrosis.

The recognition of CTGF as the common factor in driving fibrosis in various organs with fibrosis supports an import role for antagonism of CTGF for treatment of these diseases. The same group has previously shown that anti-CTGF is efficacious in mitigating fibrosis in preclinical models of neovascular age-related macular degeneration and proliferative vitreoretinopathy with minimal adverse effect on vision.^[14,16,39]^ Thus, a better understanding of the mechanisms of CTGF action and its cell autonomous activities may aid in development of new and complementary agents with effective antifibrotic activity. Recent studies have also identified the hippo signaling pathway and its downstream effectors YAP/TAZ as important modulator of CTGF production and activity.^[22]^ The YAP/TAZ inhibitor, verteporfin, suppresses CTGF production and mitigates fibrosis in the eye and heart.^[23,40,41]^ We are beginning to know more about the expression and function of CTGF using transgenic mice including CTGF reporter, and global and conditional CTGF null mice.^[2,42,43,44]^ We are learning about the role of CTGF in normal developmental and repair processes.^[43]^ A recent study targeting the deletion of CTGF in endothelial cells demonstrated an import role for CTGF-YAP axis for retinal angiogenesis and barrier formation.^[2]^ In addition, deletion of CTGF in corneal epithelial cells interfere with proper corneal wound healing.^[43]^ Thus, a better understanding of how CTGF, and perhaps other mediators, aberrant expression and downstream signaling events are regulated during early and late stages of fibrotic diseases will help to identify the common and unique regulatory mechanisms involved. We recently identified Bim expression, a proapoptotic member of Bcl-2 family, as essential for clearance of macrophages and mitigation of fibrosis in a preclinical model of choroidal neovascularization and scar formation.^[45]^ Although there are some common pathways engaged by fibrosis in different organs, there are also unique pathways which we know little about. Identification of these unique pathways and elucidation of their interactions will allow development of organ specific anti-fibrotic agents, which are tissue specific and more effective for treatment of fibrosis.

##  Financial Support and Sponsorship

None.

##  Conflicts of Interest

There are no conflicts of interest.
